# Tribological and Hardness Analyses of Friction-Stir-Processed Composites Using the Taguchi Approach

**DOI:** 10.3390/ma16010420

**Published:** 2023-01-02

**Authors:** Pragya Saxena, Arunkumar Bongale, Satish Kumar, Rangappa Suresh

**Affiliations:** 1 Symbiosis Institute of Technology, Symbiosis International Deemed University, Pune 412115, Maharashtra, India; 2 Symbiosis Centre for Applied Artificial Intelligence, Symbiosis International Deemed University, Pune 412115, Maharashtra, India; 3 Department of Mechanical and Manufacturing Engineering, M. S. Ramaiah University of Applied Sciences, Peenya, Bangalore 560058, Karnataka, India

**Keywords:** friction stir process, surface composite, Al6061, wear, hardness

## Abstract

The friction stir process (FSP) is becoming a highly utilized method to manufacture composites since it refines the microstructure and improves the physical characteristics like hardness, strength, and wear resistance of their surfaces. In this study, the hardness and wear behaviours of Al6061-based surface composites prepared by the FSP were investigated and compared for the influences of various parameters—FSP tool geometry, reinforcement composition, number of FSP passes, pin load, etc. The Taguchi design with an L27 orthogonal array was developed to analyze the influence of five input parameters on the output parameter, i.e., wear rate during wear tests. The hardness of the composite samples for different reinforcement compositions was investigated, and the results were statistically compared with the obtained wear rates. It was concluded from the results that various parameters influenced the surface wear and hardness of the composites. Tool geometries cylindrical pin and square pin had the maximum and minimum wear rates, respectively. Additionally, the optimal composition of the reinforcements copper and graphene as 1:3 possessed the maximum wear rate and minimum hardness. However, the reinforcement composition 3:3 (Cu:Gr) by weight had the minimum wear rate and maximum hardness. The higher the FSP pass numbers, the lesser the wear rate and the higher the hardness, and vice-versa. This work helps identify the influence of numerous factors on the wear and hardness aspects of surface composites prepared by the FSP. In the future, this study can be modified by combining it with thermal analysis, sensor data analysis of the composites, and optimization of the parameters for desirable microstructure and physical properties.

## 1. Introduction

Aluminium alloy-based surface composites are recently well-known as highly structural, stiff, high-strength, thermal resistant, and wear-resistant materials, particularly in sliding wear applications. These composites, having a good strength-to-weight ratio and exceptional low-temperature performance, find extensive application in various sectors of the industry like automobiles, aerospace, and manufacturing, since they attribute to high payloads (for passengers and cargo), lower fuel consumptions, lower emissions, etc. Aluminium alloys initially lack good tribological properties. Hence, their composite fabrication with suitable reinforcements and wear investigation for various parameters is critical for most industrial applications. Many researchers [1,2,3] attempted fabricated aluminium hybrid composites and studied the influence of varying process parameters (reinforcements composition, pin load, rpm of the disc, etc.) on the tribological properties of the composites. They obtained the optimal combination of the parameters for minimum wear rates. Al6061, a precipitation-hardened alloy, forms composites with superior strength and corrosion resistance, which are useful for manufacturing frames for road vehicles, railway compartments, bridges, ship buildings, towers, aircraft components, etc. Various materials are being used as reinforcements to fabricate surface composites with Al6061 alloy as the matrix material by the friction stir processing (FSP) technique. Many other researchers [4,5,6,7,8,9,10] studied Al6061-based composites using varying compositions of nano-size reinforcements and investigated their wear behaviour for different influencing parameters. Copper particles form strong intermetallic compounds with Al6061 and decrease their ductility [11]. They are an excellent in situ reinforcement with the Al6061 alloy as the matrix to form composites. However, with a large aspect ratio, graphene is lightweight and has excellent mechanical and thermal properties. It does not form intermetallic compounds with aluminium alloys and imparts its strength integral to the matrix. Adding graphene to the aluminium matrices improves the thermal conductivity and ductility of the fabricated composites. Therefore, it is a potential ex situ reinforcement for aluminium alloy-based matrices [12]. Yun Fan Dong et al. [13] found significant grain refinement of the graphene reinforcement into the aluminium matrix composites, resulting in a homogeneous distribution of particles. Many other researchers [14,15,16,17,18,19,20,21] investigated the influence of graphene reinforcement in aluminium alloy composites for their mechanical, wear, and microstructural behaviours. They obtained various process parameters affecting the composite properties. However, graphene particles may add brittleness to the matrix, so they are suitable for manufacturing surface composites as they can modify the physical properties such as hardness and resistance to wear at the surface without sacrificing the desirable characteristics of the bulk volume. Various composite fabricating techniques include diffusion bonding, powder metallurgy, plasma sintering, stir casting, etc. Many conventional methods operate at high liquefying temperatures and are used to form bulk composites, where they may cause various surface defects. Since most industrial applications include surface interactions, manufacturing surface composites has gained ample significance. Friction stir processing (FSP) is an advanced technique prominently used nowadays to fabricate surface composites. It provides an easy way to form surface composites with refined grain microstructure without involving any melting of the base material (no porosity or chemical reactions), hence improving the surface properties up to a specific depth, while the remaining volume retains its original characteristics. The invention of FSP is formed on the principle of friction stir welding (FSW), used for joining two metal plates with the help of friction caused due to high temperature caused by the tool rubbing on the workpiece surface [22].

The FSP tool geometry is an essential factor in refining the microstructure of the composites [23,24,25]. Hamidreza Eftekharinia et al. [26] investigated the wear behaviour of Al6061 alloy composite with SiC particles as reinforcements by the FSP due to tool geometry, and the number of FSP passes. They concluded that composites processed by a FSP tool with a square pin are subjected to the lowest wear, while the composites prepared by the cylindrical pin exhibit maximum wear. Reza Vatankhah Barenji et al. [27] manufactured Al6061 surface composites with Al_2_O_3_ and TiB_2_ particles as reinforcements by the FSP and studied how the pass number affects their microstructural, physical, and wear characteristics. They obtained a higher number of passes in the FSP, leading to an even dispersion of particles, high hardness, and low wear rate in the composites due to refined grain size. Recently attempted research [28] investigated the physical and wear properties of aluminium alloy composites fabricated with varying amounts of WC and Co reinforcements [29] prepared by stir casting. Microstructural analyses by SEM and XRD tests verified the homogeneous distribution of the reinforcements and the presence of oxides. Sajeeb Rahiman et al. [30] studied the influence of varying weight% of MoB, applied load at pin, and disc speed during the wear test on the tribology of Al5083. They obtained a lower wear rate when the amount of MoB weight% was increased. The experiments were designed using the Taguchi (L16) method and analysed by ANOVA to find how much the input factors influenced wear rate. It was concluded that reinforcement weight% is a major contributing factor to wear rate. Arun Premnath et al. [31] coupled the Taguchi method with the Grey method for multi-response optimization of the mechanical and wear parameters of the carbon fibre nanocomposites prepared by the hand layup method. Many researchers [32,33,34,35,36,37,38,39,40,41] investigated the physical and wear characteristics of polymer-based nanocomposites manufactured by FSP to obtain the influences of various process parameters using different optimization techniques. They found a significant rise in the composites’ flexural and tensile strengths by increasing the reinforcement content, normal load, and sliding speed up to specific limits. Some researchers [42,43,44] investigated various parameters and their influence on the properties of prepared composites using the Taguchi technique. S/N ratio plots help obtain the optimal conditions for wear loss. Sung Chan Yoo et al. [45] observed significant improvement in the properties of Al6061 alloy-based nanocomposites composed of SiC and CNT particles as reinforcements, making them suitable for heavy-duty applications. Another similar study by Shubhajit Das et al. [46] involved a wear study of an aluminium alloy composite manufactured with SiC and B_4_C particles as reinforcement. Some studies involved the study of the effects of tool rpm on the physical and tribological behaviour of the composites prepared by the friction stir process.

The fabricated composites are characterized by their physical and structural properties. Most industrial composite applications include sliding movements like pistons, control rods, valves, transmission shafts, etc., which require good wear-resistant properties. The above literature shows that the effects of wear test parameters, i.e., pin load, disc rpm, travel of pin, and reinforcement weight%, on the wear behaviour of FSP fabricated composites have been examined recently [47]. However, the influences of various parameters in the FSP, like the geometry of the tool pin, pass numbers, reinforcement composition, and weight% dispersed in the matrix, along with wear parameters on composites’ wear and mechanical behaviours, are rarely explored.

The present study is focused on examining the hardness and wear rate of Al6061 surface hybrid composites prepared by the friction stir process for varying parameters such as the geometry of the FSP tool pin, amount and composition of reinforcements, pass numbers of FSP, wear parameters, etc. The Taguchi method was utilised to design an L27 orthogonal array, and ANOVA was used to study the wear rate of the composites. Statistical analysis was used to compare the composites’ wear rate and hardness for varying reinforcement weight%.

## 2. Experimental Procedure

### 2.1. Material and Equipment

Al6061 alloy plates (180 mm × 100 mm × 10 mm) were selected as the matrix material in this study. The material composition in weight% was Mg—0.8 to 1.2, Si—0.4 to 0.8, Cu—0.15 to 0.4, Mn—0.15, Fe—0.7, Zn—0.25, and Ti—0.15. Copper powder (50 to 40 µm) and graphene nanopowder (5 to 10 nm) were selected as the reinforcements for preparing surface hybrid composites. These copper and graphene powders were mixed in a ball mill purchased from Xtreme Engineering Equipment (P) Ltd., Pune, India, in different ratios. Holes of various dimensions (3 mm and 4 mm diameters) were drilled in the matrix (Al6061) plates using HSS drill bits of respective sizes. Surface composites were manufactured by the FSP on a CNC Vertical Milling machine. The prepared composites were analyzed for hardness and wear characteristics. Wear was tested on a pin-on-disc dry sliding type wear tester. The hardness was measured for the samples at the composite surface on a Vicker’s hardness tester using a diamond indenter at a 10 kgf load. The influence of various factors was analyzed using the Taguchi approach on Minitab software.

### 2.2. Sample Preparation

**Figure 1:**
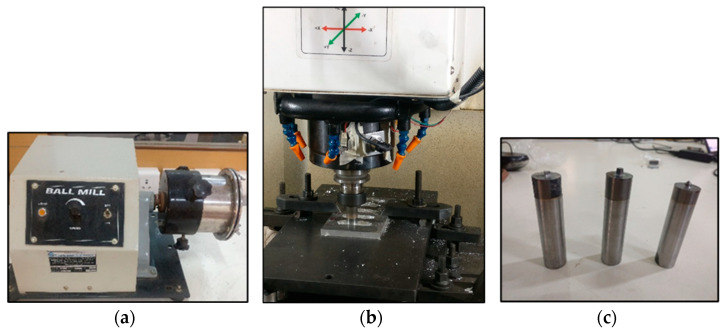
(**a**) Ball milling machine for preparing the reinforcement mixture; (**b**) FSP set up for preparing surface composites; (**c**) tools used for composite fabrication.

The Al6061 alloy plates were faced on all sides for convenient operation on the CNC milling machine. The reinforcements’ copper and graphene powder mixture was prepared in varying compositions by weight (1:1, 1:2, 1:3, 2:1, 2:3, 3:1, 3:2) according to the experimentation plan. A high-energy ball milling process was performed to prepare a uniform reinforcement mixture on a planetary ball mill (Figure 1a), available at Advanced Manufacturing Laboratory, SIT, Pune. In order to add the reinforcement mixture, 3 mm and 4 mm diameter holes were drilled in the plates.

### 2.3. Fabrication of Composites

The FSP was performed on the CNC milling machine to manufacture the Al6061 hybrid composites using specific machine parameters, i.e., tool rotation = 1000 rpm, feed = 45 mm/min, depth of cut = 5 mm (equal to pin height). The experimentation was conducted with a tungsten carbide FSP tool with different pin profiles—cylindrical, conical, square, and triangle, according to the plan designed by the Taguchi method for the wear rates of fabricated surface composites (as output response) using Minitab software. The orthogonal L27 designs were prepared for all the FSP tool pin profiles. All the surface composite samples prepared were examined. Each composite sample was prepared twice for accuracy in readings. Therefore, 216 samples were prepared in this study. Holes were drilled on each Al6061 sample for a specific quantity of reinforcement addition.

The reinforcement mixture (Cu:Gr) with different ratios (Cu:Gr = 1:1, 1:2, 1:3, 2:1, 2:3, 3:1, 3:2) was prepared by the ball milling process [48] using a planetary ball mill. The ball mill consisted of a rotating hardened steel cylindrical drum with 15–20 10 mm and 20 mm diameter ceramic coated steel balls colliding inside. This drum was rotated at 100 rpm for 5 to 10 min for uniform mixture preparation. A slurry of this mixture for each composition was prepared with ethanol solution (99%) using an agate mortar. This reinforcement slurry was poured manually into the holes on the surface of matrix plates on the Al6061 plates.

### 2.4. Wear Tests of Composites

The composite samples were prepared in specific dimensions (10 mm square base and 20 mm height) according to the machine configuration and ASTM G99 standards for wear tests on the pin-on-disc sliding type tribometer. These samples were fixed in a 10 mm × 10 mm square size sample holder one by one for wear tests at room temperature. While conducting the wear tests, the sample was held on a sample holder fixed inside an arm supported by a pulley system so that the sample face touched the disc (EN31 steel). Load was attached to the pulley system, which acted on the sample resting over the disc by a lever mechanism. The test of the prepared samples was conducted keeping the track diameter constant, i.e., 80 mm, pin load in the range of 30 N, 40 N, and 50 N, and the disc speed in the range of 400, 500, and 600 rpm with a constant duration of 20 min. Table 1 shows the wear test input factors and their levels as selected for the wear tests of the samples.

**Figure 2:**
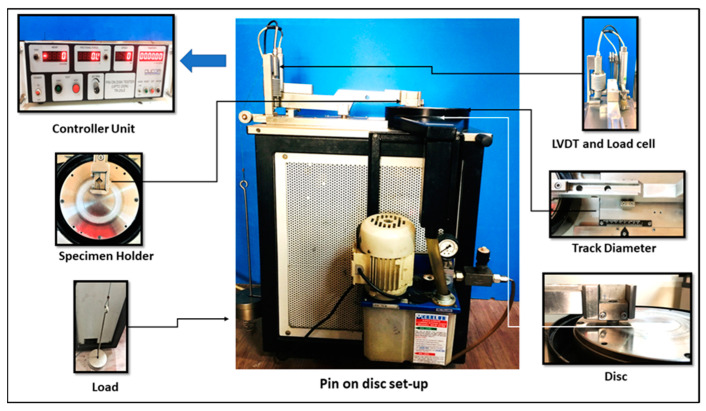
Pin-on-disc wear test setup.

The pin-on-disc setup (Figure 2) consisted of two sensors, a linear variable differential transformer (LVDT), and a friction sensor. These sensors, mounted on the machine, were connected to the controller and monitor attachment to display the wear measurement (in micrometres) and the frictional force (in N) during the wear examination. The wear measurement and friction force values were displayed in graphs with time using Winducom 2010 software, version 1.0, purchased by Ducom Instruments Pvt. Ltd., (Bengaluru, India), on the monitor. The weights of the samples before and after the tests were measured using a precision electronic weight balance machine with an accuracy of ±0.0001 g.

#### Taguchi’s Design of Experiments

The composite samples prepared (by Tools A, B, C, and D) were tested for their wear behaviour by the wear test as conducted on a pin-on-disc wear tester according to the experimental plan designed by the Taguchi method (L27 orthogonal array) on Minitab software. It is a powerful technique to investigate the effects of multiple input factors on desired output responses individually and interactively. This method reduces the time, cost, and the number of experiments for the desired outcome without compromising the accuracy and quality of results. Table 2 shows the design of experiments for composites prepared by FSP Tool A, i.e., cylindrical pin. Five input factors, i.e., copper weight%, graphene weight%, number of passes, pin load, and disc speed, were investigated for three levels on the output response as wear rate. The wear rates of the samples were calculated according to the following equation:

$$\mathrm{Wear}\;\mathrm{Rate}=\frac{\mathrm{Mass}\;\mathrm{of}\;\mathrm{the}\;\mathrm{specimen}\;\mathrm{lost}}{\left(\mathrm{Sliding}\;\mathrm{Distance}\right)\times \left(\mathrm{Time}\;\mathrm{Duration}\right)}\;=\frac{\mathrm{Mass}\;\mathrm{of}\;\mathrm{the}\;\mathrm{specimen}\;\mathrm{lost}}{\left(\Pi \;\times \;\mathrm{Track}\;\mathrm{diameter}\;\times \;\mathrm{Disc}\;\mathrm{speed}\;\times \;\mathrm{Time}\;\mathrm{duration}\right)\times \left(\mathrm{Time}\;\mathrm{Duration}\right)}$$

### 2.5. Hardness Tests of Composites

The hardness of the composite samples was tested for the composite samples prepared for varying composition of the reinforcement on a Vicker’s hardness testing machine, and the results are tabulated with comparison to the wear results in Table 3. In this experiment, a diamond indenter with an angle of 136° was forced into the workpiece surface to form an indent. This indenter was fixed to the holder and advanced vertically downwards onto the sample surface at the selected load (10 kgf). Vicker’s hardness number was calculated as:

$$\mathrm{HV}=\frac{\mathrm{Force}\;\mathrm{Applied}}{\mathrm{Area}\;\mathrm{of}\;\mathrm{indentation}}=\frac{1.8544\;F}{d²}$$

## 3. Results and Discussion

### 3.1. Wear Test Analysis

#### 3.1.1. Effect of Tool Pin Geometry

**Figure 3:**
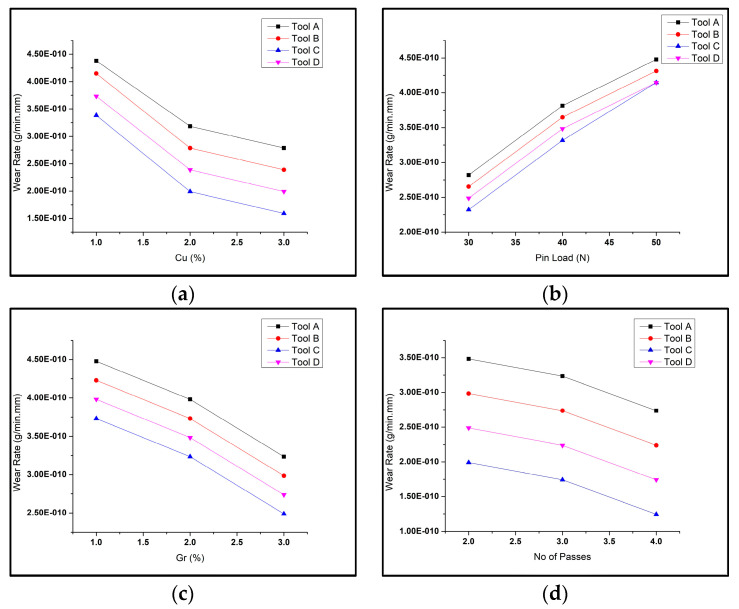
(**a**) WR vs. copper weight% at 500 rpm, 30 N; (**b**) wear rate (WR) vs. pin load at 2% copper, 600 rpm; (**c**) WR rate vs. graphene weight% at 400 rpm, 50 N; (**d**) WR vs. number of passes at 400 rpm, 30 N, for Tools A, B, C, D.

The variation in wear rate values in composites for varying parameters for different tool geometries is shown in Figure 3. For all the graphs, it can be noted that for all conditions, the wear rate was maximum for Tool A, i.e., the cylindrical pin profile, and was minimum for Tool C, i.e., the square pin profile. This study confirms the results illustrated in previous literature [26]. The reason is that when a square pin rotates inside the matrix surface, it causes rapid variation in material flow, leading to dynamic loading, fragmentation, and homogeneous distribution of the reinforcement particles (refined in size) on the composite surface. Additionally, the trend of the wear rate curves was similar for the FSP by all of the pin profiles. It was visible that on increasing the pin load, the wear rate increased with a higher rate for 30 N to 40 N and rose slowly from 40 N to 50 N. It was interpreted that the wear rate reduced with an increase in reinforcement weight% in the composite.

#### 3.1.2. Effect of Reinforcement Weight%

**Figure 4:**
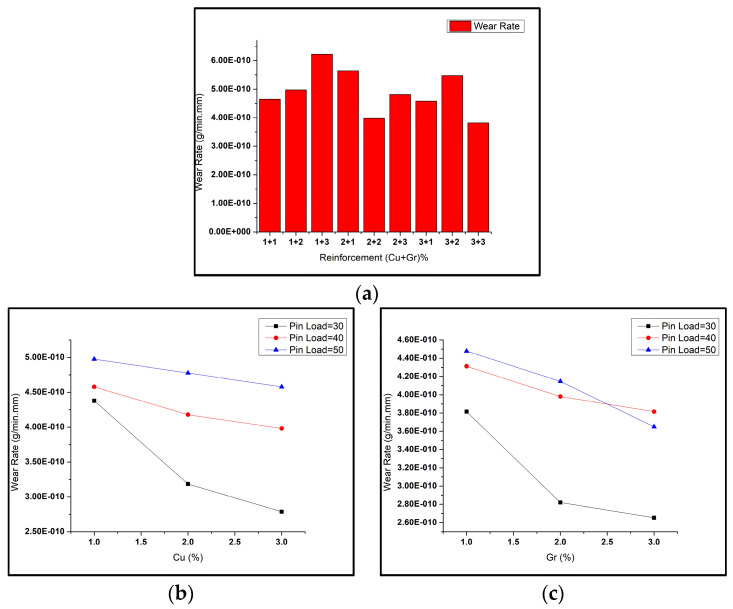
(**a**) Wear rates of composites with varying reinforcement weight% (copper and graphene); (**b**) WR vs. copper weight% at 500 rpm; (**c**) WR vs. graphene weight% at 600 rpm.

Different combinations of reinforcement weight% (copper and graphene) were used to prepare the surface hybrid composites. Al6061 alloy matrices, being soft, undergo adhesive wear, exhibiting high wear rates. However, adding copper and graphene powders as reinforcement into these matrices resists the applied load and prevents material loss, resulting in reduced wear rates [28]. The variation in wear rate of the samples for various combinations by weight of copper (Cu) and graphene (Gr) particles is shown in Figure 4. It can be seen that the amount and composition of the reinforcements added caused considerable effects on the wear rate of tested samples. The wear tests caused a massive reduction in the size of the particles of the reinforcement mixture as fabricated by the FSP [49]. With higher reinforcement (weight%), the particles on the composite surface acted as a solid lubricant film on the composite surface, reducing the delamination caused by wear. For the composite samples with reinforcements Cu:Gr at 3:3, the wear rate value obtained was the lowest, while for composites with reinforcements Cu:Gr at 1:3, the wear rate value obtained was maximal. In the figures, the wear rate of the tested composites was visible as maximal for the highest pin load (i.e., 50 N) and was minimal for the lowest pin load (i.e., 30 N). With a rise in copper weight%, it was visible that the wear rate decreased. Hence, copper addition improved wear resistance in the Al6061 composite. An increase in graphene weight% (from 1% to 3%) caused a reduction in the wear rate of the composite [50]. However, the trend varied for different pin load conditions.

#### 3.1.3. Effect of Pin Load

**Figure 5:**
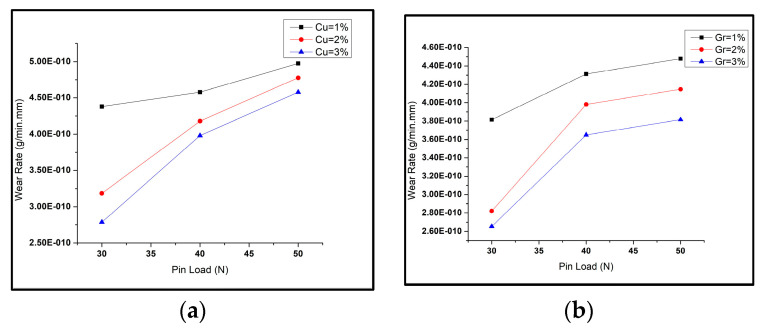

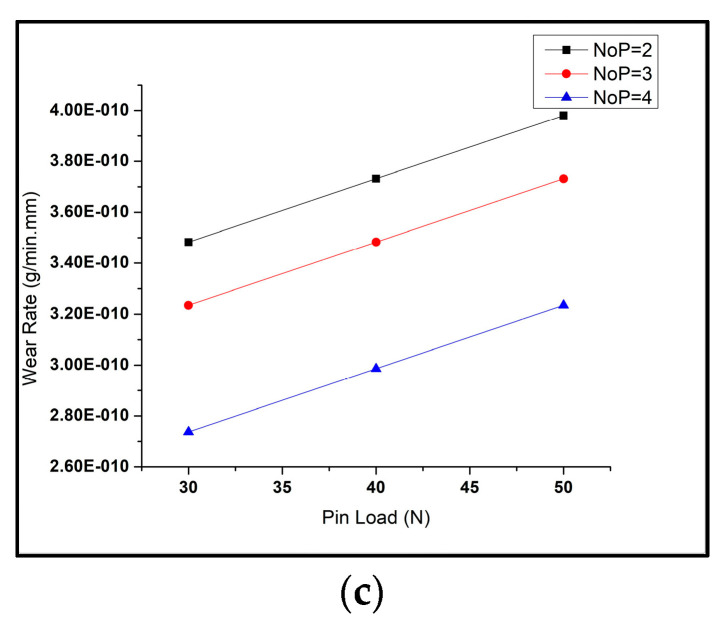
(**a**) Wear rate with varying pin load for different Cu% at 500 rpm; (**b**) wear rates with varying pin load for different Gr% at 600 rpm; (**c**) wear rate with varying pin load for different number of FSP passes.

The wear rate values of samples with varying pin loads for different parameters kept constant, is shown in Figure 5. As the pin load increased, the wear rate of the composite rose. From Figure 5a,b, it is clear that the wear rate increased with the rise in pin load for different copper weight% and graphene weight%. In Figure 5c, the wear rate appears to rise with an increase in load at pin for various FSP passes.

#### 3.1.4. Effect of the Number of Passes

**Figure 6:**
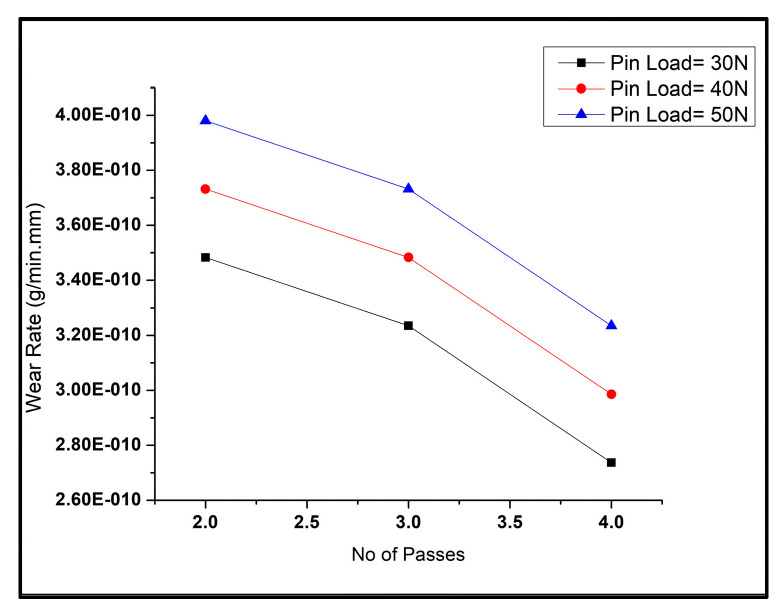
Wear rates of composites with varying number of passes.

The graph in Figure 6 shows that the pass numbers undertaken by the tool during the process highly influenced the wear rate of the tested samples. The variation of wear rate with the change in the number of passes during the FSP for different pin loads is visible in Figure 6. It was inferred from the graph that with a rise in pass numbers, the wear rate decreased. An even refined grain dispersion of reinforcement, i.e., copper and graphene particles, was obtained after every pass. This grain refinement led to reduced surface defects such as porosity, clusters of particles, etc. It helped increase the contact surface area between the composite sample and the rotating disc while reducing the wear rate with every pass.

#### 3.1.5. ANOVA of Wear Tests

The wear experiments of the composite samples were conducted on a pin-on-disc tester according to the run order given by the Taguchi method, and the wear results for the tested composites, manufactured by FSP Tool A, are explained in this section. In this work, the five input factors’ (copper weight%, graphene weight%, number of FSP passes, pin load, and disc speed) influence on the wear rate was obtained for three levels (g/min.mm) of samples during wear tests using Minitab software.

**Figure 7:**
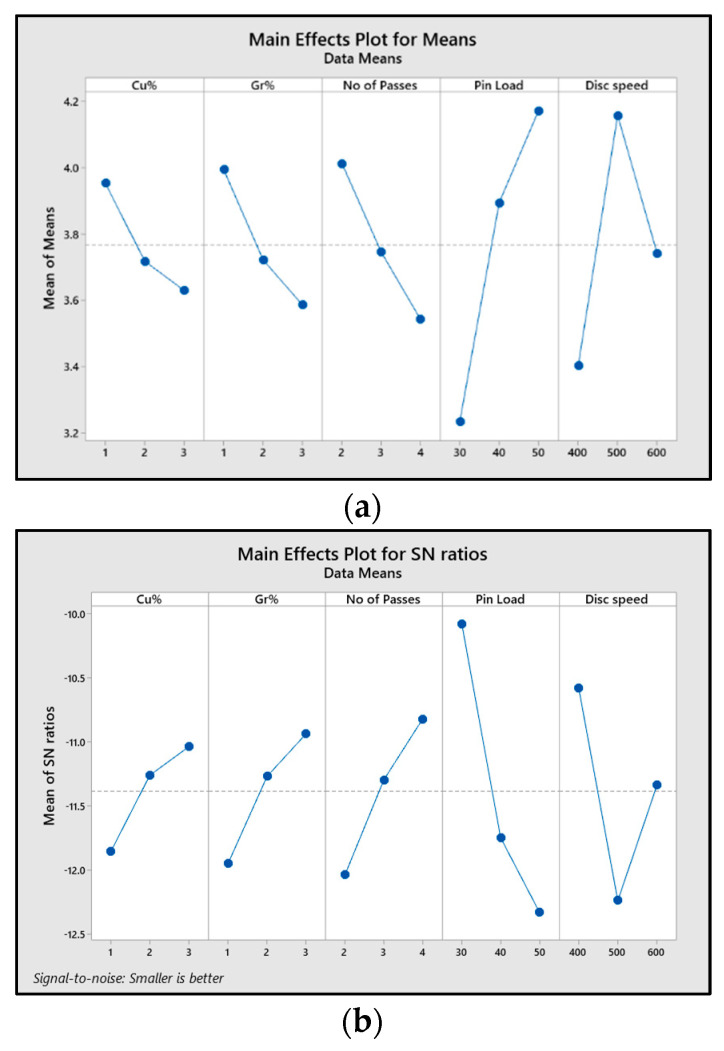
Main effect plot for (**a**) mean values of wear rate and (**b**) SN ratios of wear rate for varying parameters (Cu%, Gr%, No of passes, pin load, disc speed).

The main effect plots for means and “signal-to-noise ratio” (SN ratio) variation of the “wear rate” (g/min.mm) of the composite on the influence of varying input parameters are shown in Figure 7a,b. The S/N ratio plots determine the optimal values of all the input parameters influencing the output response. It was inferred that wear rate values fell as the copper weight% increase from 1% to 3%. The reason for the decrease in wear rate was the strong intermetallic compounds formed by copper with the Al6061 matrix, which added wear resistance to the surface of the composite. Similarly, adding graphene powder (1 to 3 by weight%) as ex situ reinforcement on the matrix surface increased the hardness. It reduced the wear rate of the matrix surface without any compound formation with the matrix. Therefore, 3% copper and 3% graphene were the optimal values for minimal wear rate. The graphs show the fall in wear rates as the number of FSP passes increased from two to four. The reason was the refined grain size and even dispersion of particles on the composite surface with every pass. Therefore, to obtain the lowest wear rate, four FSP passes were the optimal value. With the rise in pin load (from 30 to 50 N) and disc speed (from 400 to 600 rpm), the wear rate increased due to high dynamic loading and more material removal caused by rubbing the composite surface with the disc, respectively. Hence, the optimal values of pin load and disc speed were 30 N and 400 rpm, respectively, to obtain the lowest wear rate values. Hence, the optimal conditions for the lowest wear rate were obtained from the main effect plots as A_3_, B_3_, C_3_, D_1_, and E_1_.

**Figure 8:**
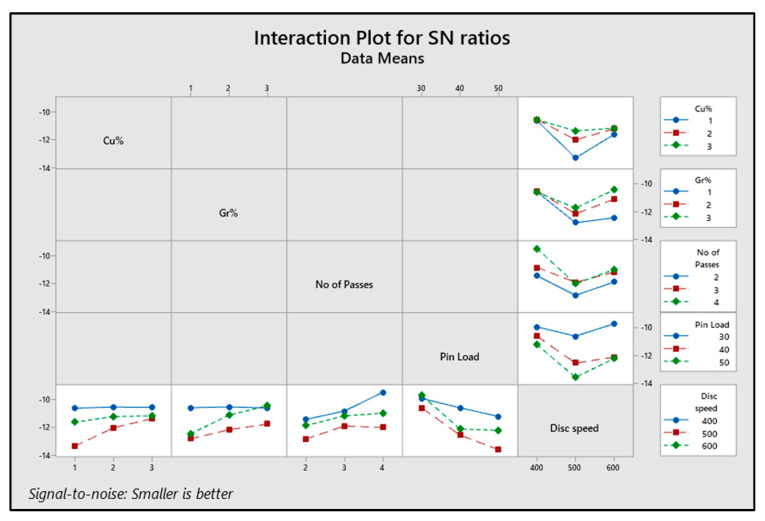
Interaction plot for the SN ratios of wear rate for varying parameters (Cu%, Gr%, No of passes, pin load, disc speed).

Figure 8 shows the interaction plot for the study that included the influence of all the factors on the output response (wear rate) interactively.

##### Significant Factors Affecting Wear Rate

The significance of the parameters influencing the wear rate was evaluated by an ANOVA using Minitab software. ANOVA was implemented at a significance level of 5% and a confidence level of 95% to obtain the most influential parameter on the output response. The optimal values of the parameters were obtained from a signal-noise-ratio analysis for the “smaller-the-better” characteristic.

The ANOVA, as shown in Table 4, depicted the factors affecting the tribology of composites processed by Tool A. Here, the degree of freedom was obtained by subtracting one from the level of each factor considered during the experiments. The *p*-value in the table indicates the measure of evidence against the null hypothesis, which rejects it if it is lesser than the significance level. The last column for the contribution factor (%*p*) was added to obtain the most influential parameter, which was calculated by obtaining the percentage of Adj SS, i.e., adjusted sum of squares. The contribution factor indicates how much influence is contributed by that factor. It is inferred that %*p* for pin load had a maximum value, and hence it most significantly affected the wear rate of the composites. Additionally, the least significant factor was copper weight%. The model summary for the performance parameter R^2^ for the ANOVA for all the composites prepared by all the tools, i.e., Tools A, B, C, and D, are shown in Table 5. It is visible that the value of R^2^ for composites processed by Tool C was maximal, showing more significance of influencing parameters.

The response table for S/N ratios of all input factors affecting the output parameter is shown in Table 6. The rank order indicates the pattern in which all the parameters affect the output response. The table shows that the delta value (distinction in maximal and minimal values) for pin load was the highest; hence, it had the maximal effect on the wear rate of samples. Similarly, copper weight% was the least affecting parameter.

- Multiple linear regression:

By implementing the regression analysis, the wear rate for the composites was predicted based on all the input factors and their significance obtained from ANOVA. Minitab software was used to develop an empirical linear model to predict the wear rates on the influence of all the input parameters. The predicting equations were as shown below:

Regression Equations

For Tool A

Wear Rate × 10^ − 10 = 2.473 − 0.162 Cu% − 0.205 Gr% − 0.235 No of Passes + 0.0470 Pin Load + 0.00170 Disc speed

For Tool B

Wear rate × 10^ − 10 = 1.003 − 0.075 Cu% − 0.095 Gr% − 0.084 No of Passes + 0.0721 Pin Load + 0.00041 Disc speed

For Tool C

Wear rate × 10^ − 10 = −2.049 − 0.054 Cu% − 0.047 Gr% − 0.018 No of Passes + 0.0955 Pin Load + 0.00272 Disc speed

For Tool D

Wear rate × 10^ − 10 = −0.474 − 0.069 Cu% − 0.071 Gr% − 0.051 No of Passes + 0.0838 Pin Load + 0.00147 Disc speed

In the regression model predicted by the software, the value of R^2^, i.e., coefficient of determination, was obtained as a measure of variability in values of one factor that could be caused due to its interdependence with other related factors. This method examined the model’s potential to predict wear rate for new observations. For example, if we put the values of all the factors for a particular experimental run in the model, we get the estimated wear rate slightly different from the one we got from the experiments. This deviation of the fit value from the experimental result is called the residual error. The R^2^ value here was obtained as 79.98% and 73.86% for the composites processed by Tool C and Tool D, respectively, which is a good fit with some residual errors or variability in comparison to that for composites processed by Tools A and Tool B. The optimal wear rates for all the cases can be obtained by putting the optimal values (as obtained from the SN ratios analysis) of all the parameters in the respective regression equations.

**Figure 9:**
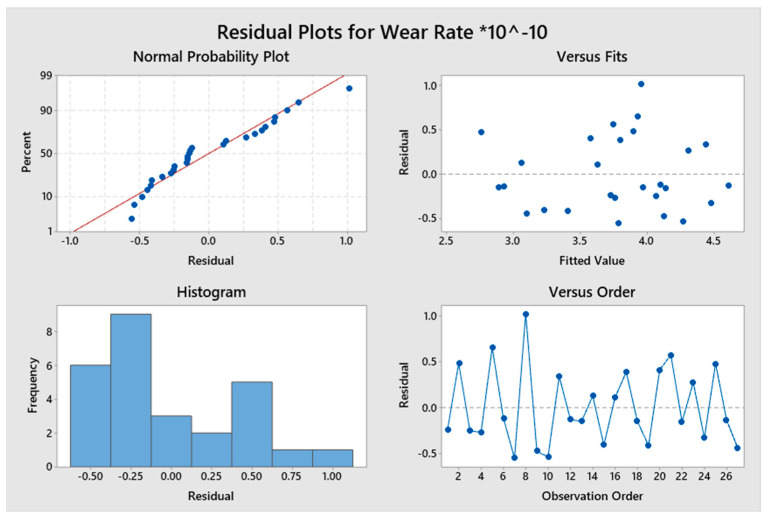
Residual plot for the wear rate for varying parameters.

Figure 9 shows the residual plots, which indicated the consistency of all the experimental data for the composites processed by Tool A. Significance of the coefficient was predicted by the residual plots. The word residual means the error. The distance of the dots to the probability line is called the error. The normal probability plots for the residuals indicate that data points are adjacent to the trend line. The error bars or the residuals were more concentrated in the lower region, which suggests that the obtained results were precise. The residuals in the graph were scattered randomly above and below the zero axis, showing that they possessed constant variance. It can be estimated from the graphs that the residuals or the errors were within the control range, indicating the model’s sufficiency.

### 3.2. Analysis of Hardness

Vicker’s hardness test setup was utilised to capture the hardness of composite samples. The average hardness of the friction-stir-processed Al6061 alloy with no reinforcements was obtained as 72.14188734 HV. The influence of FSP tool geometry, amount of reinforcement added, and pass number in the FSP on hardness values of the samples were investigated in the following subsections.

#### 3.2.1. Effect of Tool Geometry

**Figure 10:**
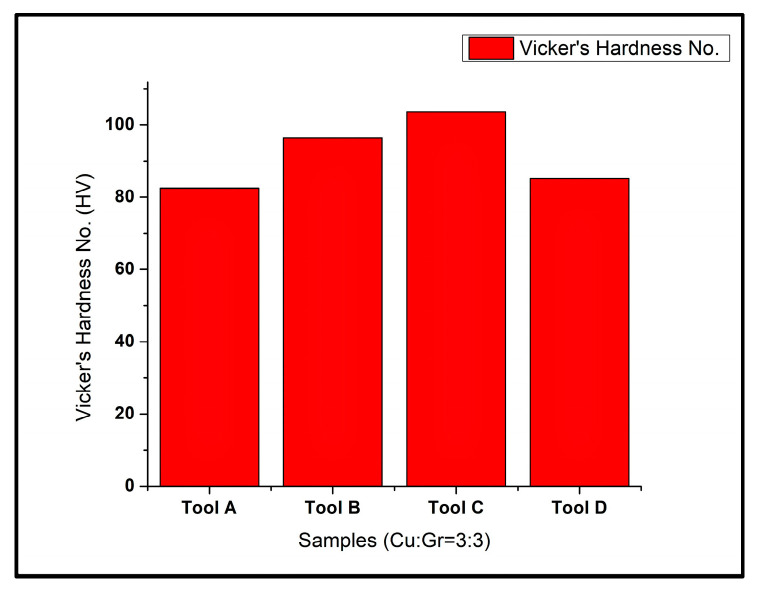
Vicker’s hardness number (HV) of the composites prepared by Tools A, B, C, D.

The hardness profile for the processed composites by different geometries of the tools is shown in Figure 10. It is visible that the hardness profile of the composite sample prepared by FSP tools varied significantly for different tool pin geometries (cylindrical, conical, square, and triangular). The composite samples manufactured by the cylindrical pin tool (Tool A) were found to have minimal hardness, while the composites prepared by the square pin FSP tool (Tool C) had the highest hardness values. The reason for high hardness in the case of the square profile was the fluctuation in the area stirred by the flat-faced tool pin inside the flowing material, resulting in refined grain size and even dispersion of particles. However, the cylindrical pin stirred the material uniformly, and the particles accumulated with the tool pin, causing a coarser dispersion of reinforcement particles and the formation of clusters inside the material surface. Hence, the lowest hardness values were obtained in the composite samples prepared by the cylindrical pin tool.

#### 3.2.2. Effect of Reinforcement Weight%

**Figure 11:**
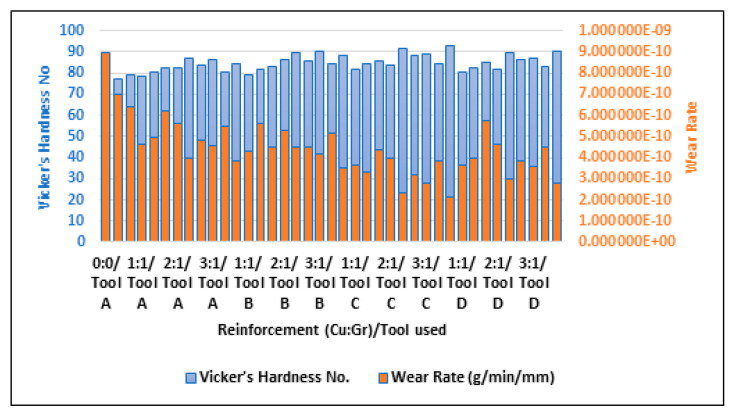
Comparison of wear rate and Vicker’s hardness number (HV) of the composites.

Figure 11 shows the comparison of wear rate and Vicker’s hardness number for varying compositions of reinforcement (copper and graphene). It is inferred that if a surface has a high hardness value, it will have good wear resistance and hence will give a lower value for wear rate when tested. From Table 3, the sample with no reinforcement (Cu:Gr = 0:0) had the lowest value of Vickers’ hardness and the highest value of wear rate. Similarly, the composite samples with copper and graphene at 3:3 (3 weight% each) had the highest hardness and lowest wear rates for composites processed by all the tools of all tested geometries.

#### 3.2.3. Effect of Number of Passes

**Figure 12:**
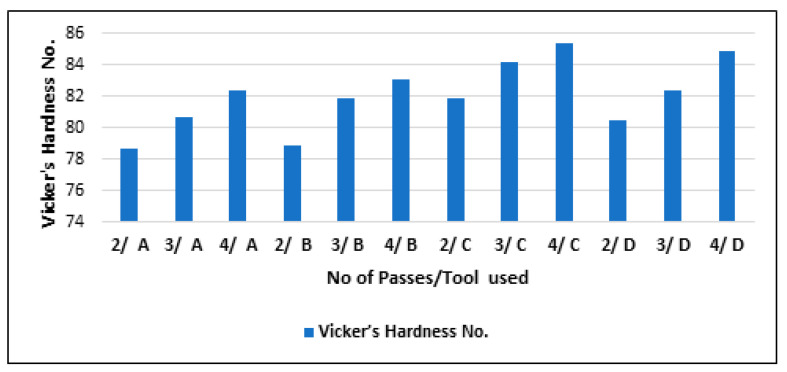
Vicker’s hardness number (HV) of the composites processed for a varying number of FSP passes.

The hardness values of the composites varying with a different number of passes (two, three, and four) for all the tool geometries (A, B, C, and D) is shown in Figure 12. It is visible in the graph that as the pass number increased, the composites’ hardness also increased. The increased hardness was due to the refined grain size dispersion obtained in the stir zone with every pass. After two passes, the mean size of the particles in the processed composite samples was higher. Hence, hardness values were the lowest. Nonetheless, after four passes, the grains were refined to a much-reduced size, so the composites’ hardness was maximal.

## 4. Conclusions and Future Scope

The wear rate and the hardness of the Al6061 hybrid surface composite samples prepared using the FSP technique using four tool pin profiles were analyzed for varying parameters using statistical and Taguchi ANOVA. Based on the studies, some important conclusions were obtained as follows:

- The wear rate was maximal and hardness was minimal in composite fabrication by the FSP using Tool A, i.e., the cylindrical pin profile. In contrast, the minimal wear rate and maximal hardness were obtained for the samples processed by Tool C, i.e., the square pin profile. The reason for the high hardness and low wear rate of the composites prepared using Tool C was the rapid variation in the material flow leading to dynamic loading, refined grain size, and uniform distribution of the particles when the square pin stirred inside the material.
- It was verified from the results of the experiments that if a surface had a high hardness value, it also had good wear resistance and hence exhibited a lower wear rate value.
- Addition of copper and graphene (1% to 3% both) as in situ and ex situ reinforcements, respectively, to the Al6061 matrix, refined the grain size and improved the wear resistance and hardness of the composites. The lowest wear rate and highest hardness values were obtained for the Al6061-based composites for copper and graphene particles at 3:3, whereas the wear rates were maximal and hardness was minimal for the composites with Cu:Gr at 1:3.
- From the wear experiments, it was verified that the wear rate increased with the rise in pin load and disc speed. The reason was excessive rubbing on the surface when the load at the pin and rotational speed of the disc were increased.
- When the composite was prepared by the FSP technique, it was concluded that with a higher number of passes, the microstructure was more refined, and the reinforcements were more uniformly dispersed. Therefore, for a higher number of FSP passes, the wear rate was reduced, hardness was increased, and vice-versa.
- From ANOVA with wear rate as the output response and five input factors (pin load, Cu%, Gr%, No of passes, disc speed), pin load was the most significant parameter affecting the wear rate, while copper weight%. was the least influencing factor on wear rate.
- The regression analysis for wear rate prediction of the composites (prepared by Tools A, B, C, D) was based on all the input factors and their significance obtained from ANOVA. It was concluded from all four models that the model was a good fit for composites prepared by Tool C, followed by Tools D, B, and A, respectively.

Composites fabrication and their mechanical and tribological characterization for varying FSP and wear test parameters were performed in this study. This study can be modified by collecting sensor data, such as temperature, vibration, forces, etc., during composite fabrication and analyzing that sensor data to obtain the conditions of the composites fabricated for varying parameters by the FSP process using suitable machine learning algorithms. The study can also be improved to predict and classify the faults caused during composite fabrication and relate them to the possible causes of their occurrence. As a result, the modification of parameters in order to improve the microstructure and physical as well as wear properties of the composites may be implemented.

## Figures and Tables

**Table 1:** Pin-on disc wear test input factors and levels.

S.No.	Reinforcement Weight%		No of FSP Passes	Pin Load	Disc Speed
	Cu%	Gr%			
1	1	1	2	30	400
2	2	2	3	40	500
3	3	3	4	50	600

**Table 2:** Taguchi’s experiments designed for wear tests of the composites prepared by Tool A.

S.No.	Cu%	Gr%	No of Passes	Pin Load	Disc Speed	Weight Loss (g)	Wear Rate (g/min.mm)
1	1	1	2	30	400	0.014	3.48 × 10^−10^
2	1	1	2	30	500	0.022	4.38 × 10^−10^
3	1	1	2	30	600	0.023	3.81502 × 10^−10^
4	1	2	3	40	400	0.014	3.48328 × 10^−10^
5	1	2	3	40	500	0.023	4.58 × 10^−10^
6	1	2	3	40	600	0.024	3.98089 × 10^−10^
7	1	3	4	50	400	0.013	3.23447 × 10^−10^
8	1	3	4	50	500	0.025	4.97611 × 10^−10^
9	1	3	4	50	600	0.022	3.65 × 10^−10^
10	2	1	3	50	400	0.015	3.73 × 10^−10^
11	2	1	3	50	500	0.024	4.77707 × 10^−10^
12	2	1	3	50	600	0.027	4.4785 × 10^−10^
13	2	2	4	30	400	0.011	2.73686 × 10^−10^
14	2	2	4	30	500	0.016	3.18 × 10^−10^
15	2	2	4	30	600	0.017	2.8198 × 10^−10^
16	2	3	2	40	400	0.015	3.73209 × 10^−10^
17	2	3	2	40	500	0.021	4.17994 × 10^−10^
18	2	3	2	40	600	0.023	3.81502 × 10^−10^
19	3	1	4	40	400	0.012	2.98567 × 10^−10^
20	3	1	4	40	500	0.02	3.98 × 10^−10^
21	3	1	4	40	600	0.026	4.31263 × 10^−10^
22	3	2	2	50	400	0.016	3.98089 × 10^−10^
23	3	2	2	50	500	0.023	4.58 × 10^−10^
24	3	2	2	50	600	0.025	4.14676 × 10^−10^
25	3	3	3	30	400	0.013	3.23447 × 10^−10^
26	3	3	3	30	500	0.014	2.78662 × 10^−10^
27	3	3	3	30	600	0.016	2.65393 × 10^−10^

**Table 3:** Vicker’s hardness number compared to the wear rate for the samples.

S.No.	Reinforcement (Cu: Gr)/Tool Used	Number of Passes	D1 (mm)	D2 (mm)	Vicker’s Hardness No.	Wear Rate (g/min/mm)
1	0:0/Tool A	2	0.502	0.512	72.14188734	8.957010 × 10^−10^
2	0:1/Tool A	2	0.492	0.489	77.07710516	6.985670 × 10^−10^
3	1:0/Tool A	2	0.485	0.482	79.32506959	6.378980 × 10^−10^
4	1:1/Tool A	2	0.482	0.489	78.67286213	4.644400 × 10^−10^
5	1:2/Tool A	3	0.481	0.478	80.65405287	4.976100 × 10^−10^
6	1:3/Tool A	4	0.472	0.477	82.36277775	6.220100 × 10^−10^
7	2:1/Tool A	3	0.476	0.473	82.36277775	5.639600 × 10^−10^
8	2:2/Tool A	4	0.464	0.461	86.69218408	3.980900 × 10^−10^
9	2:3/Tool A	2	0.469	0.474	83.41420682	4.810200 × 10^−10^
10	3:1/Tool A	4	0.461	0.465	86.50504504	4.578000 × 10^−10^
11	3:2/Tool A	2	0.478	0.481	80.65405287	5.473700 × 10^−10^
12	3:3/Tool A	3	0.471	0.468	84.12638465	3.815000 × 10^−10^
13	1:1/Tool B	2	0.487	0.483	78.83515783	4.312630 × 10^−10^
14	1:2/Tool B	3	0.473	0.479	81.84450251	5.639600 × 10^−10^
15	1:3/Tool B	4	0.473	0.472	83.06150444	4.478500 × 10^−10^
16	2:1/Tool B	3	0.464	0.463	86.3185113	5.307860 × 10^−10^
17	2:2/Tool B	4	0.458	0.452	89.57372298	4.478500 × 10^−10^
18	2:3/Tool B	2	0.462	0.467	85.94724932	4.478500 × 10^−10^
19	3:1/Tool B	4	0.455	0.451	90.36640693	4.179940 × 10^−10^
20	3:2/Tool B	2	0.471	0.468	84.12638465	5.141990 × 10^−10^
21	3:3/Tool B	3	0.461	0.457	88.0193278	3.483280 × 10^−10^
22	1:1/Tool C	2	0.479	0.473	81.84450251	3.649150 × 10^−10^
23	1:2/Tool C	3	0.468	0.471	84.12638465	3.317400 × 10^−10^
24	1:3/Tool C	4	0.463	0.469	85.39483137	4.379000 × 10^−10^
25	2:1/Tool C	3	0.469	0.473	83.59140105	3.980900 × 10^−10^
26	2:2/Tool C	4	0.452	0.448	91.57530864	2.322200 × 10^−10^
27	2:3/Tool C	2	0.463	0.455	88.0193278	3.151500 × 10^−10^
28	3:1/Tool C	4	0.459	0.455	88.79142347	2.819800 × 10^−10^
29	3:2/Tool C	2	0.469	0.467	84.66652056	3.815000 × 10^−10^
30	3:3/Tool C	3	0.449	0.446	92.60135451	2.156300 × 10^−10^
31	1:1/Tool D	2	0.482	0.478	80.48611111	3.649150 × 10^−10^
32	1:2/Tool D	3	0.476	0.473	82.36277775	3.980890 × 10^−10^
33	1:3/Tool D	4	0.468	0.467	84.84772227	5.772290 × 10^−10^
34	2:1/Tool D	3	0.474	0.478	81.84450251	4.644370 × 10^−10^
35	2:2/Tool D	4	0.457	0.453	89.57372298	2.985670 × 10^−10^
36	2:3/Tool D	2	0.468	0.46	86.13258026	3.815020 × 10^−10^
37	3:1/Tool D	4	0.464	0.46	86.87993104	3.582800 × 10^−10^
38	3:2/Tool D	2	0.474	0.472	82.88599153	4.478500 × 10^−10^
39	3:3/Tool D	3	0.454	0.451	90.56622203	2.819800 × 10^−10^

**Table 4:** Analysis of variance (ANOVA) for wear rate of the composites prepared by FSP Tool A.

Source	DF	Adj SS	Adj MS	F-Value	*p*-Value	%*p*
Cu%	2	0.5061	0.2530	1.83	0.192	4.484634743
Gr%	2	0.7812	0.3906	2.83	0.089	6.922340765
No of Passes	2	1.0001	0.5001	3.62	0.051	8.86204941
Pin Load	2	4.1961	2.0980	15.18	0.000	37.1823273
Disc speed	2	2.5905	1.2953	9.37	0.002	22.95484351
Error	16	2.2112	0.1382			
Total	26	11.2852				

**Table 5:** Model summary for ANOVA table for Tools A, B, C and D.

Tool	S	R-sq	R-sq (adj)	R-sq (pred)
A	0.371755	80.41%	68.16%	44.20%
B	0.350984	86.74%	78.46%	62.25%
C	0.351629	91.13%	85.58%	74.74%
D	0.345158	89.37%	82.72%	69.73%

**Table 6:** Response table for S/N ratios for wear rate of composites processed by Tool A. Smaller is better.

Level	Cu%	Gr%	No of Passes	Pin Load	Disc Speed
1	−11.86	−11.95	−12.04	−10.08	−10.58
2	−11.26	−11.27	−11.30	−11.75	−12.24
3	−11.04	−10.94	−10.82	−12.33	−11.34
Delta	0.82	1.01	1.22	2.26	1.66
Rank	5	4	3	1	2

## Data Availability

The data of the experiment are available from the authors.
